# To Stop Bleeding

**Published:** 1880-01

**Authors:** 


To Stop Bleeding, if from a cavity in the jaw after a tooth has been extracted, shape a cork into the proper form and size to cover the bleeding cavity, and long enough to be kept firmly in place when the mouth is closed. This, we believe, is our own invention, and we have never known it to fail. It has served us in desperate cases.

When an Artery is Cut, the red blood spurts out at each pulsation. Press the thumb firmly over the artery, near the wound, and on the side toward the heart. Press hard enough to stop the bleeding, and wait till a physician comes. The wounded person is often able to do this himself, if he has the requisite knowledge. The main object of this "Journal" is to teach its readers, not only how to protect themselves from diseases, but how to save life in accidents and emergencies.

Simple Fractures may be adjusted by almost any one. Get the limb as near as possible in the natural position, and then send for a doctor. There is no great urgency in such cases.

In Fracture of the Skull, with compression and loss of consciousness, examine the wound, and, if possible, raise the broken edges of the skull so as to relieve the pressure on the brain. Prompt action would often save life.

In Case of Poisoning, the simple rule is to get the poison out of the stomach as soon as possible. Mustard and salt act promptly as emetics, and they are always at hand. Stir a tablespoonful in a glass of water, and let the person swallow it quickly. If it does not cause vomiting in five minutes, repeat the dose. After vomiting, give the whites of two or three eggs, and send for the doctor.

Burns and Scalds are soonest relieved by an application of cold water. Dry carbonate of soda, or baking soda, sprinkled over the burned spot, is the latest remedy, and is said to be very effectual. These means are only temporary. In severe cases, a physician should be sent for.

